# Cytotoxicity of Probiotics from Philippine Commercial Dairy Products on Cancer Cells and the Effect on Expression of *cfos* and *cjun* Early Apoptotic-Promoting Genes and *Interleukin-1* *β* and *Tumor Necrosis Factor-α* Proinflammatory Cytokine Genes

**DOI:** 10.1155/2014/491740

**Published:** 2014-09-07

**Authors:** Peter T. Shyu, Glenn G. Oyong, Esperanza C. Cabrera

**Affiliations:** Biology Department and Center for Natural Sciences and Ecological Research, De La Salle University, 2401 Taft Avenue, 1004 Manila, Philippines

## Abstract

This study determined cytotoxicity of probiotic* Lactobacillus* spp. from Philippine dairy products on cancer cells and normal fibroblasts and their effects on expression of early apoptotic-promoting* cfos, cjun* and proinflammatory cytokine* IL-1* *β*,* TNF-α* genes. Cultures were from Yakult, Bear Brand Probiotic Drink, Nido3+ Powdered Milk. Filter-sterilized supernatants from cultures of* Lactobacillus* spp. were evaluated for cytotoxicity to colon cancer cells (HT-29 and HCT116), leukemia cells (THP-1), and normal human dermal fibroblasts (HDFn) using PrestoBlue. Bleomycin was the positive control. Absolute quantification of transcript levels was conducted using qRT-PCR. Cytotoxicity index profiles on HDFn, THP-1 of all probiotic supernatants and negative controls suggest nontoxicity to the cells when compared to bleomycin, whereas all probiotic supernatants were found to be cytotoxic to HT-29 and HCT-116 colon cancer cell lines. Expression of* cfos, cjun* transcripts was significantly upregulated in HT-29 and HCT116 cells treated with probiotic supernatants compared to untreated baseline levels (*P* < 0.05). Expression of* IL-1* *β* and* TNF-α* by lipopolysaccharide-treated macrophages was significantly downregulated in cells with probiotic supernatants compared to those exposed to MRS medium (*P* < 0.05). Results provide strong support for the role of* Lactobacillus* spp. studied in anticancer therapy and in prevention of inflammation that may act as precursor to carcinogenesis.

## 1. Introduction

In recent years, cancer has been reportedly increasing in terms of both incidence and mortality rates in less developed countries, with colon cancer as the third most frequent form of cancer in males and fourth in females [1, 2]. On the other hand, leukemia has been found to compromise immune-competence in patients, further leading to an increased susceptibility to nosocomial bacterial infections, which in turn negatively affects the therapeutic outcome [3].

Among several other distinct features, cancer cells are characterized by an acquired insensitivity to growth inhibitory signals, as well as to signals that would normally induce apoptosis, or programmed cell death [4]. This may be associated with an alteration in the mitogen-activated protein kinase (MAPK) pathway that regulates the processes of cell division and cell death. The MAPK signal transduction pathway results in the formation of the AP-1 transcription factor, a heterodimer protein composed of the gene products of the* cfos *and* cjun* genes. AP-1 has been reported to be involved in cell apoptosis [5], and it was reported that the activation of the* cfos* and* cjun* components of the AP-1 transcription factor increases sensitivity of prostate cancer cells to the tumor necrosis factor related apoptosis inducing ligand (TRAIL) pathway of apoptosis by negative regulation and repression of the antiapoptotic molecule c-FLIP(L) [6]. Several studies have also supported the role of the protein products of* cfos *and* cjun *in inducing cell death as a result of cell trauma, exposure to antitumor drug, or withdrawal of survival signals and growth factors [7–11].

Inflammation is a defense mechanism of the body in response to injury caused by infection and harmful stimuli. Immediate resolution of inflammation following infection is termed as acute inflammation and is beneficial for counteracting infection and cell injury [12]. However, failure to resolve inflammation due to changes in the normal immune response may lead to chronic inflammation and alterations in cancer-related genes, which may contribute to tumor formation and metastasis [13]. Some of the effects of inflammation that may contribute to carcinogenesis include causation of genomic instability, epigenetic alterations leading to altered gene expression, enhanced proliferation and invasiveness of cells, and resistance to apoptosis [12]. Moreover, chronic inflammation caused by infections, autoimmune diseases, and infections of uncertain origins have been associated with 25% of cancers worldwide [14].* Tumor Necrosis Factor-*α* (TNF-*α*)* and* Interleukin-1*β* (IL-1*β*)* are two among the various cytokines produced primarily by activated macrophage cells in response to inflammatory stimuli. Aside from their proinflammatory functions, several studies have also reported increased levels of these cytokines in some types of cancer, thereby providing strong support for their possible roles in cancer progression [15–17].

Recently, a growing number of studies have increasingly shown the various roles of probiotics and have highlighted the many health benefits of the latter. Different microbial species belonging to the genus* Lactobacillus* have been of particular interest in the development of probiotics.* Lactobacillus *belongs to a group of Gram-positive, heterotrophic, nonmotile, and nonsporulating bacteria known as lactic acid bacteria (LABs), which are characterized by their production of lactic acid. Aside from being associated with the gastrointestinal tract, LABs are also known as additives to various food products, such as milk and meat products. More notably, several studies have reported on the role of LABs, specifically those belonging to the genus* Lactobacillus*, against carcinogenesis. Some species that have been identified to be anticancer agents include* Lactobacillus rhamnosus, L. casei, L. bulgaricus, *and* L. acidophilus *[18, 19].

However, despite the increasing works of literature on the promising effects of the* Lactobacillus *spp. against carcinogenesis, majority of the studies conducted have placed their focus only on colon carcinoma cells. Little is known about their effect on leukemia. Moreover, the mechanisms by which* Lactobacillus* spp. induce cytotoxicity on cancer cells and their role in the induction of anti-inflammatory responses have not been fully understood, despite the many studies that support the relationship between the latter and cancer formation [18, 20, 21].

This study aimed to provide information on potential mechanisms by which probiotic* Lactobacillus* spp. can induce selective cytotoxic, proapoptotic effects on colon and leukemia cancer cell lines, as well as anti-inflammatory effects on macrophage cells at the molecular level. Furthermore, since conventional methods of cancer treatment are known to cause nonspecific cytotoxic effects on human cells, as well as several other adverse effects on the health of cancer patients, the study aimed to contribute to the development of alternative methods of cancer therapy utilizing probiotic* Lactobacillus *spp. currently available and accessible to the general public in dairy products, which would yield the same, if not greater, potency of treatment without the risk of compounded health problems. Given the growing body of evidence that associates inflammation with carcinogenesis, this study also aimed to contribute to the development of anti-inflammatory agents that would aid in the prevention of the latter.

## 2. Materials and Methods

**Figure 1:**
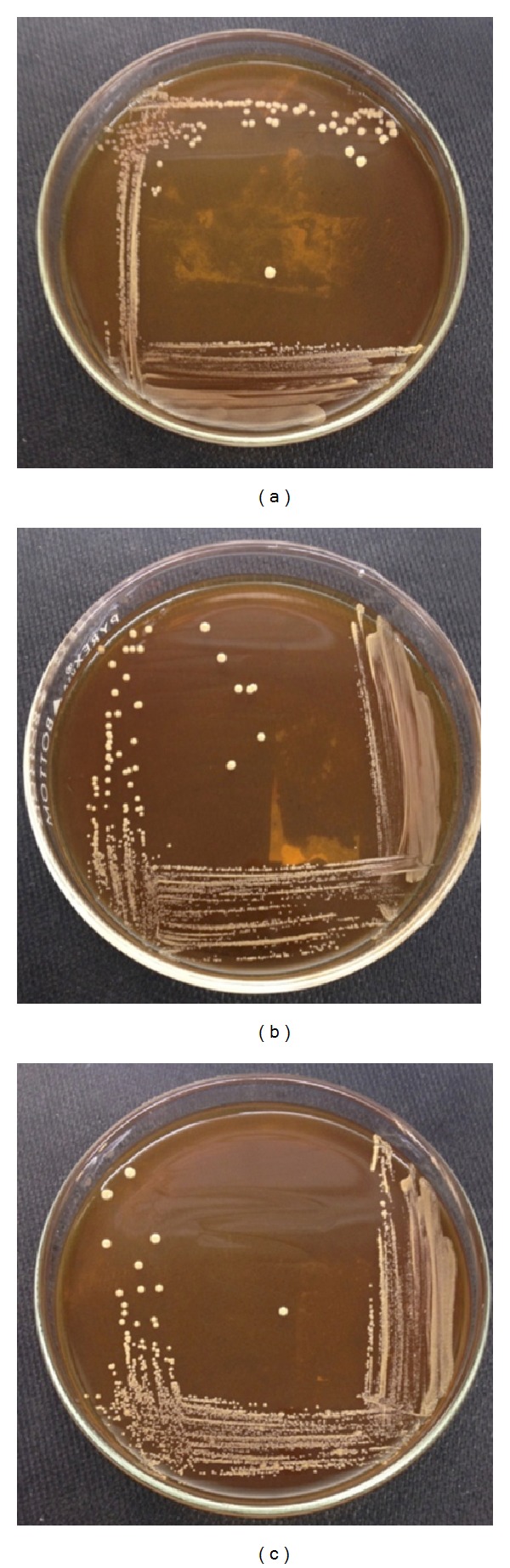
Isolated colonies of stock probiotic* Lactobacillus *spp. from (a) Bear Brand, (b) Nido, and (c) Yakult cultured in deMan, Rogosa, and Sharpe (MRS) agar medium after incubation for 24 hours at 37°C and 5% CO_2_.

**Figure 2:**
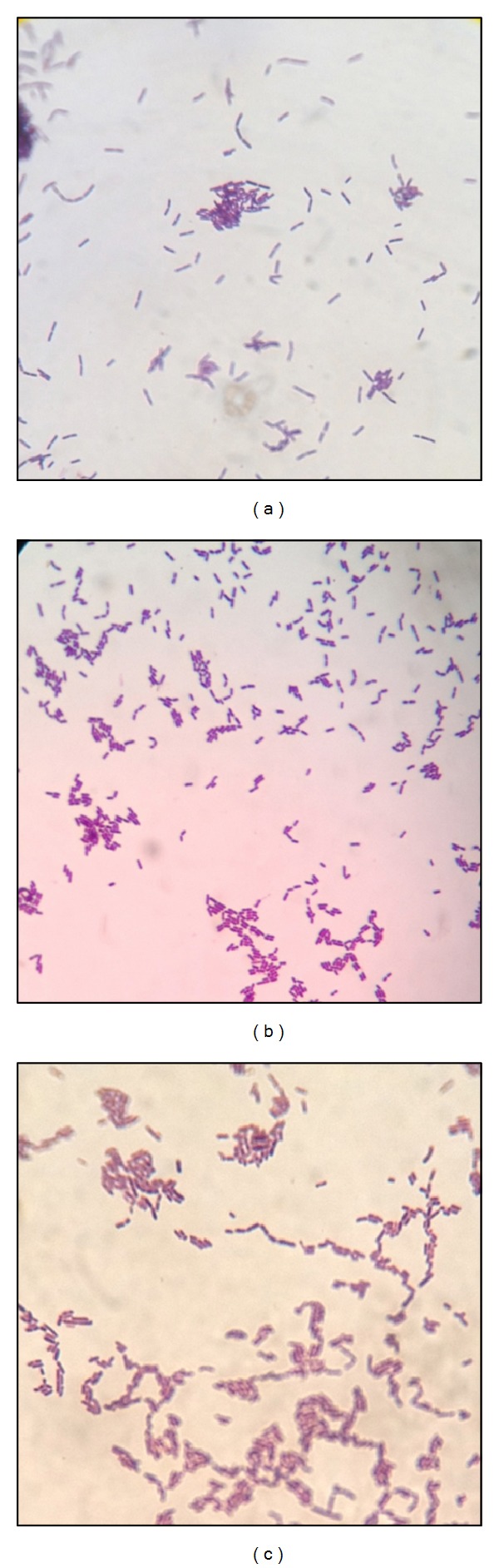
Gram stain results of* Lactobacillus* spp. from (a) Bear Brand, (b) Nido, and (c) Yakult showing Gram (+) nonspore-forming bacilli.

*Lactobacillus *spp. were previously isolated by Shi [22] from three commercially available milk products, namely, Yakult, Nido 3+, and Bear Brand (see Figures 1 and 2). Colon cancer cell line HT-29, leukemia cell line THP-1, and normal fibroblast HDFn were generously provided by the Center for Natural Science and Ecological Research (CENSER) of De La Salle University, Manila, Philippines. The colon cancer cell line HCT 116 is a kind gift from Dr. Sonia D. Jacinto of the Institute of Biology, University of the Philippines-Diliman. All cell lines were cultured in 50 mL T-flasks (Falcon, USA), and all incubation processes were done under the following conditions: 5% CO_2_ with 95% humidity at 37°C.

Bacterial isolates from the three dairy product sources were grown in deMan, Rogosa, and Sharpe (MRS) broth at 37°C with 5% CO_2_ for 48 hours. The optical densities of the resulting bacterial cultures were adjusted to correspond to OD_550 nm_ of 1.03 (3 × 10^8^ CFU/mL) [19, 23], and the tubes were centrifuged at 13,000 rpm for 3 minutes. The clear supernatant of each culture was carefully obtained and filter-sterilized using a 0.45 *μ*m syringe filter (Millipore, USA) and was used to test for cytotoxicity. The pH of the cell-free supernatants and the uninoculated MRS broth that was used as the negative control were also determined.

HT-29, HCT116, and HDFn (Invitrogen, USA) cells were cultured in Dulbecco's Modified Eagle Medium (DMEM) (Gibco, USA) with the addition of 10% fetal bovine serum or FBS (Gibco, USA) and 1x antimycotic antibiotic (Gibco, USA) as a monolayer. HT-29, HCT116, and HDFn were independently harvested with 0.05% trypsin-EDTA in phosphate-buffered saline (PBS; pH 7.4). THP-1 cells were cultured in Roswell Park Memorial Institute or RPMI (Gibco, USA) medium supplemented with 10% FBS, 1x antimycotic antibiotic.

### 2.1. Cell Viability Assay

The following procedure was adapted from the study conducted by Rezaei et al. [24] and Oyong et al. [25] with some modifications.

All cell lines were cultured to 90% confluence, harvested, and separately seeded in 100 *μ*L volume per well in a 96-well culture plate (Corning, USA). Each 100 *μ*L inoculum had a density of 2.4 × 10^5^ viable cells mL^−1^ as determined using the Trypan Blue Exclusion method. The plates were incubated for six (6) days. After attachment of cells to the bottom of the wells as monolayers, 100 *μ*Ls of twofold dilutions of each of the three (3) prepared filter-sterilized culture supernatants and the MRS broth were added into each well. Twofold serial dilutions of the anticancer drug bleomycin (1 U/mL) were used as the positive control, and DMEM broth with 10% FBS for the untreated setup served as the negative control. All five (5) treatments were done in triplicate, and the plates were incubated for 72 hours.

After incubation, 10 *μ*L of PrestoBlue (Invitrogen, USA) was added to each well. The plates were incubated for another four (4) hours. A microplate reader (BioTek ELx800, BioTek Instruments, USA) was used for colorimetric detection of the reduction of resazurin to resorufin through absorbance measurements taken at 570 nm. The resulting cytotoxicity index values were subsequently plotted against the concentration of the different probiotic supernatants to obtain the corresponding linear equations for the calculation of the minimum inhibitory concentration (IC_50_) for each probiotic supernatant.

### 2.2. *cfos* and* cjun* Transcript qRT-PCR Assay for Early Apoptosis

HT-29 and HCT116 cells, to which the probiotic supernatants demonstrated cytotoxicity, were further tested for the expression of the early apoptosis genes,* cfos* and* cjun*, as follows.

One hundred *μ*L of HT-29 and HCT116 cells (2.4 × 10^5^ cells/mL) was separately seeded into 96-well plates and incubated for 24 hours. After attachment of the cells as monolayers, volumes corresponding to the IC_50_ of the three probiotic supernatants and of bleomycin were added into each well. Bleomycin was used as the positive control, while the negative control included only cancer cells and cancer cells treated with MRS broth. Each setup was done in duplicate. After 30 minutes of treatment exposure, total RNA was extracted from the cells using the TriZol Reagent (Invitrogen, USA).

All qRT-PCR reactions were carried out using the Rotor-Gene 3000 thermocycler in a final volume of 10 *μ*L solution containing 1 *μ*L of the RNA template, 5 *μ*L of 2x KAPA FAST SYBR (KAPA Biosystems, USA), 0.3 *μ*L each of the 10 *μ*M forward and reverse primers for both* cfos* and* cjun*, and 3.4 *μ*L of Diethylpyrocarbonate- (DEPC-) treated water (Invitrogen, USA). The primer sequences are as follows: F: 5′- AAGGAGAATCCGAAGGGAAAGGAATAAGATGGCT-3′, R: 5′-AGACGAAGGAAGACGTGTAAGCAGTGCAGCT-3′ for* cfos*, and F: 5′-GCATGAGGAACCGCATTGCCGCCTCCAAGT-3′, R: 5′-GCGACCAAGTCCTTCCCACTCGTGCACACT-3′ for* cjun* [26].

Initial cDNA synthesis step was done at 50°C for 3 minutes, followed by cDNA amplification consisting of 40 cycles of 95°C for 20 seconds, 55°C for 30 seconds, and 72°C for 35 seconds. Melting analysis was done from 72 to 95°C.

Quantification of amplified transcript levels was done with respect to the simultaneously amplified human glyceraldehyde-3-phosphate dehydrogenase (GAPDH) internal standard concentrations of different known magnitudes, namely, 10^9^, 10^8^, 10^7^, 10^6^, and 10^5^ copies. This was done through the use of the Rotor-Gene 3000 software ver. 6.1.93, which plots a standard curve from which critical threshold (Ct) values were derived.

### 2.3. *IL1-* *β* and* TNF-α* Transcript qRT-PCR Assay for Anti-Inflammatory Activity

The anti-inflammatory assay of the culture supernatants was adapted from Majlesi [27] with modifications and was conducted on THP-1 cells. A volume of 100 *μ*L of THP-1 cells (2.4 × 10^5^ cells/mL) was seeded into the wells of a 96-well culture plate, added with 2 *μ*L of phorbol myristate acetate (PMA) (Sigma Aldrich, USA), and incubated for 48 hours to induce differentiation into macrophages. One hundred microliters of 100 ng/mL lipopolysaccharide or LPS (Sigma Aldrich, USA) was then added, and the plates were incubated for one hour. The subsequent experimental setup done is described as follows.

The six (6) negative controls consisted of wells containing 100 *μ*L with 2.4 × 10^5^ cells/mL of THP-1 or PMA-treated THP-1 cells. These are as follows: (1) THP-1 cells cultured to 90% confluence in DMEM medium with 10% FBS, (2) PMA-induced THP-1 cells, (3) PMA-induced THP-1 cells and MRS broth used in the preparation of probiotic cultures, and (4–6) PMA-induced THP-1 cells and the volume of the spent probiotic media corresponding to their respective IC_40_ values.

The positive control consisted of PMA-induced THP-1 cells added with 100 *μ*L of 100 ng/mL LPS after their differentiation into macrophages [28]. The treatment setup consisted of THP-1 cells treated with PMA, LPS, and volumes of culture supernatants corresponding to IC_40_ values.

Plates were incubated for one (1) hour after the addition of LPS before administration of the probiotic treatments. All setups were done in duplicate. Incubation time was 30 minutes before the addition of 30 *μ*L RNA Later reagent (Invitrogen, USA) to preserve the RNA integrity of the samples prior to RNA extraction using the TriZol Reagent.

All qRT-PCR reactions were done using the Rotor-Gene 3000 thermocycler in a final volume of 10 *μ*L solution containing 1 *μ*L of the RNA template, 5 *μ*L of 2x KAPA FAST SYBR (KAPA Biosystems, USA), 0.3 *μ*L each of the 10 *μ*M forward and reverse primers for* IL1-* *β* and* TNF-α*, and 3.4 *μ*L of Diethylpyrocarbonate- (DEPC-) treated water (Invitrogen, USA). The primer sequences are as follows: F: 5′-ATGAAGTGCTCCTTCCAGGACCTG-3′, R: 5′-CCTGGAGTGGAGAGCTTCAGTT-3′ for* IL-1* *β*, and F: 5′-GGACGTGGAGCTGGCCGAGG-3′, R: 5′-TGGGAGTAGATGAGGTACAGGCCC-3′ for* TNF-α* [27].

PCR conditions were as follows: initial cDNA synthesis was done at 50°C for 3 minutes, followed by cDNA amplification consisting of 40 cycles at 95°C for 20 seconds, 50°C for 40 seconds, and 72°C for 20 seconds. Completion of the profile was done by melting from 72 to 95°C using the Corbett RG-3000. Quantification of amplified transcripts was done with reference to established 5-magnitude concentrations of human GAPDH cDNA internal standard.

### 2.4. Statistical Analysis

Significant differences between the different treatments are determined using Student's Paired *t*-test performed on Microsoft Excel. A probability value of *P* < 0.05 was designated as the reference value to indicate a statistical difference between treatment results.

## 3. Results and Discussion

**Figure 3:**
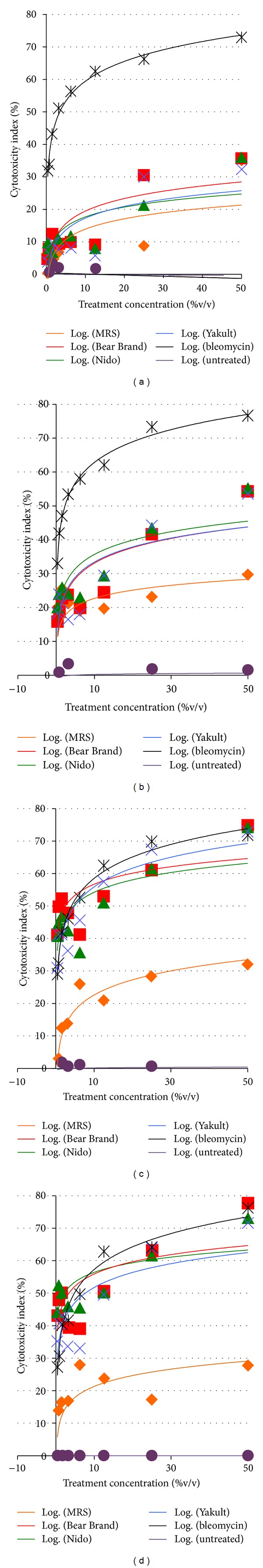
Cytotoxicity index (CI%) plots of (a) HDFn, (b) THP-1, (c) HT-29, and (d) HCT116 cells after 72-hour exposure to the different probiotic supernatant treatments, deMan Rogosa, and Sharpe (MRS) broth medium and the anticancer drug bleomycin.

**Figure 4:**
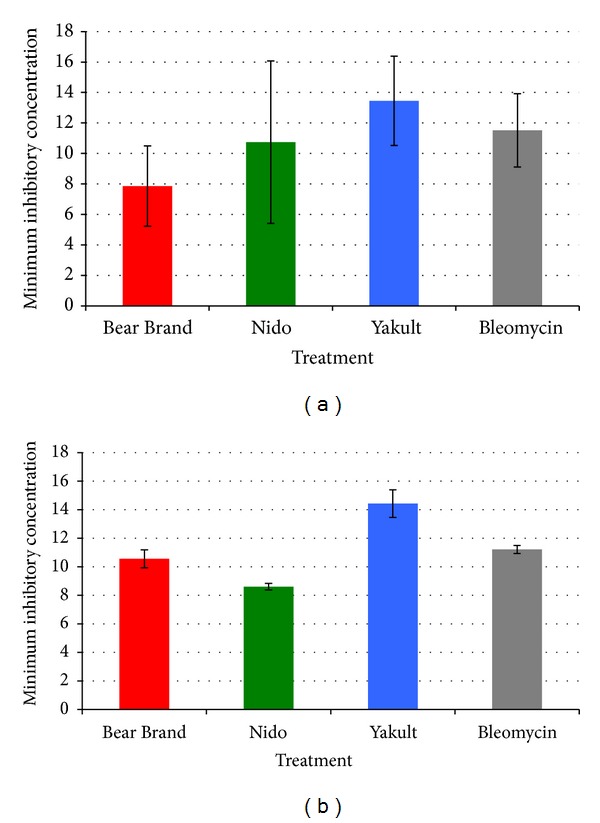
IC_50_ values of the different probiotic treatment groups and the anticancer drug bleomycin on the (a) HT-29 and (b) HCT116 colon cancer cell lines. No significant difference was found between the probiotic treatment groups and the bleomycin for both cell lines (*P* > 0.05).

The cytotoxicity of the probiotic supernatants on the four cell lines based on the cell viability assay is shown in Figures 3 and 4. Based on the obtained cytotoxicity index (CI%) plots, the IC_50_ of the anticancer drug bleomycin was shown to be 9.31% for HDFn and 4.77% for THP-1 cells. No significant cytotoxic activity was observed with the spent media supernatants of all* Lactobacillus *spp. and the MRS broth medium on both HDFn and THP-1 cell lines as there was no identifiable probiotic treatment concentration that would inhibit at least 50% of the total number of cells (i.e., no IC_50_ value was determined) (Figures 3(a)-3(b)).

Conversely, all probiotic supernatant treatments showed significant and specific cytotoxicity against the HT-29 and HCT116 cell lines, which was similar to that of bleomycin (Figures 3(c)-3(d)). It could be noted that the Bear Brand treatment group had a higher cytotoxicity for HT-29 cells (IC_50_ = 7.86%) compared to that of bleomycin (IC_50_ = 11.49%) (Figure 4(a)), while the Nido treatment had a higher cytotoxicity (IC_50_ = 8.47%) compared to bleomycin (IC_50_ = 11.34%) for HCT116 cells (Figure 4(b)). However, no significant difference was found between the two groups for either cell line (*P* > 0.05). The same is true between bleomycin and the other two probiotic treatment groups for both HT-29 and HCT116 cells.

**Figure 5:**
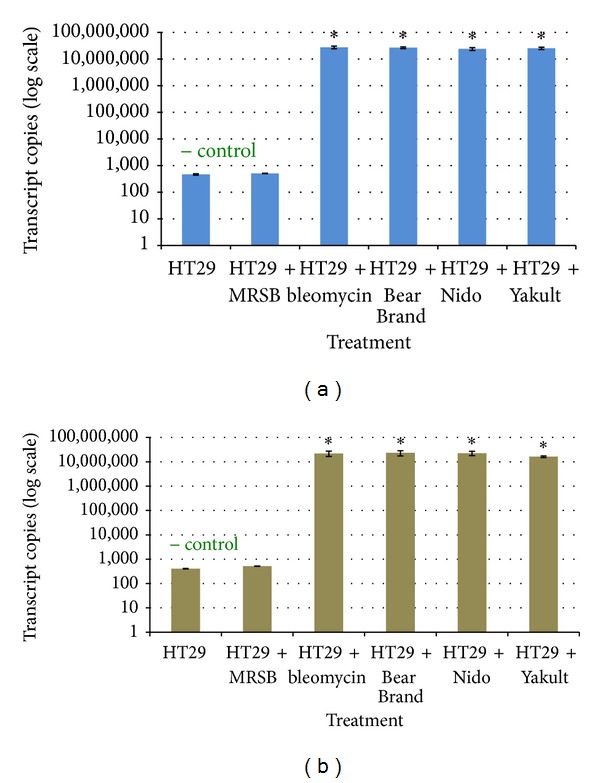
qRT-PCR plots of the transcript copy numbers of the early apoptosis markers (a)* cfos *and (b)* cjun* genes from treated and untreated HT-29 cells. Significant difference from the negative control is indicated by an asterisk ∗.

**Figure 6:**
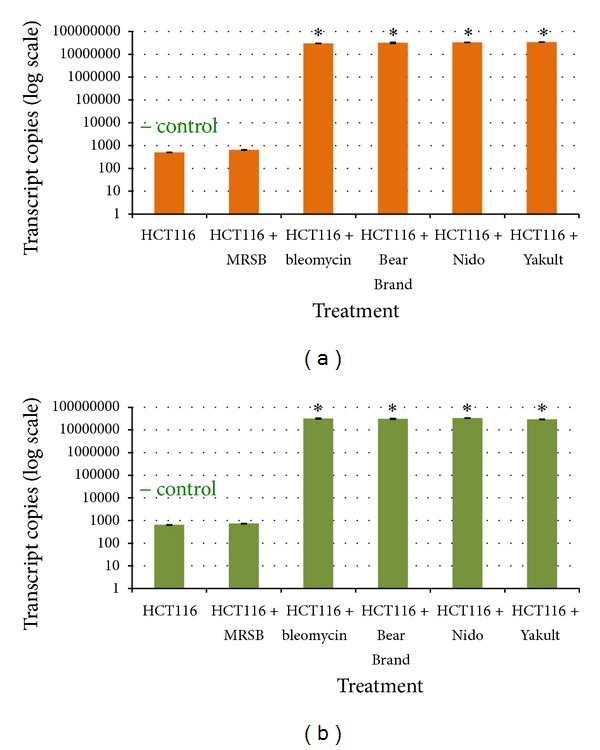
qRT-PCR plots of the transcript copy numbers of the early apoptosis markers (a)* cfos *and (b)* cjun *genes from treated and untreated HCT116 cells. Significant difference from the negative control is indicated by an asterisk ∗.

The expression of the early apoptosis marker gene* cfos *was significantly increased in HT-29 cells treated with Bear Brand (*P* = 0.001), Nido (*P* = 0.003), and Yakult (*P* = 0.002) probiotic supernatants (Figure 5(a)) compared to the untreated negative control and cells treated with MRS broth. The same trend was observed for the expression of* cjun*, which was also significantly increased in the Bear Brand (*P* = 0.014), Nido (*P* = 0.001), and Yakult (*P* = 0.002) treatment groups (Figure 5(b)). A significant increase in the expression of both* cfos* and* cjun* was also observed for HCT116 cells treated with the three probiotic supernatants (*P* < 0.05) (Figures 6(a)-6(b)).

**Figure 7:**
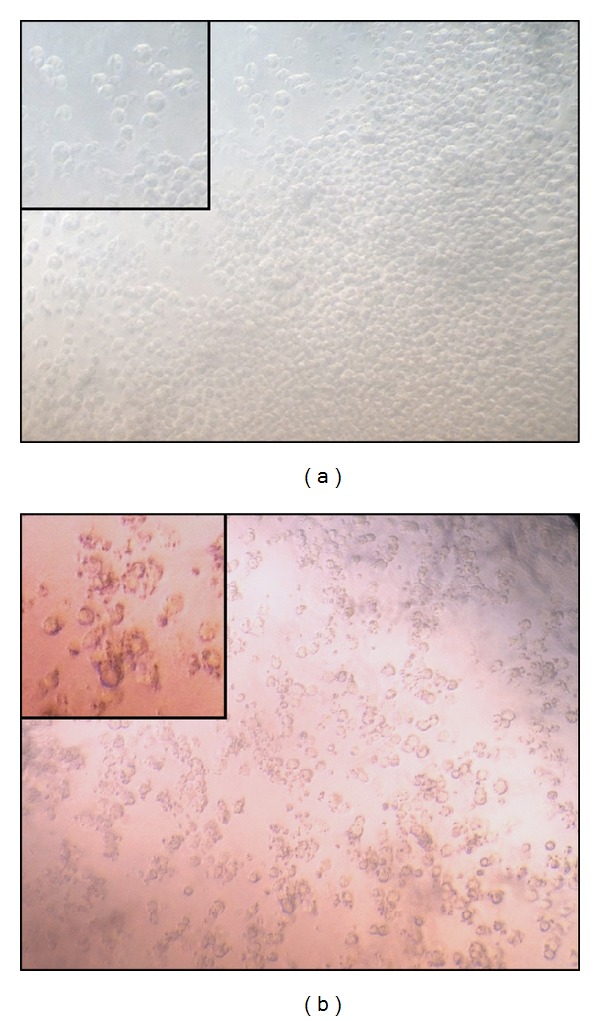
Photomicrograph of (a) THP-1 undifferentiated monocytes and (b) phorbol myristate acetate- (PMA-) differentiated macrophages (100x). Cut-in images show differences in cellular morphology (150x).

**Figure 8:**
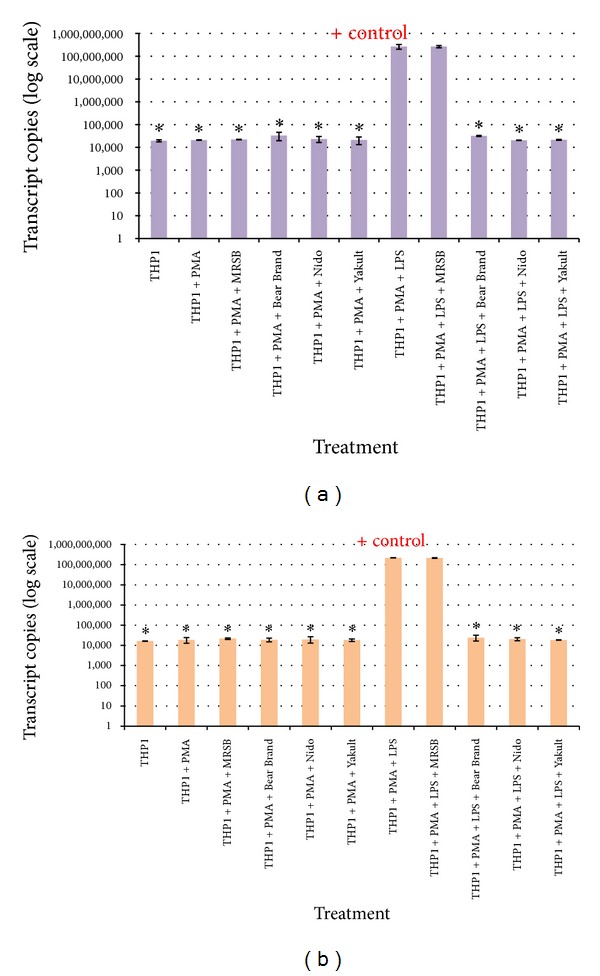
qRT-PCR plots of the transcript copy numbers of the (a)* IL1-* *β* and (b)* TNF-α*cytokine genes. Significant difference from the control group positive for inflammation is indicated by an asterisk ∗.

Macrophages differentiated from THP-1 cells after exposure to the bacterial antigen LPS were used to evaluate* IL1-* *β* and* TNF-α* expression (Figure 7).  Figure 8 shows the downregulation of the expression of both proinflammatory cytokine genes. The downregulation in cells exposed to any of the three probiotic supernatants was significantly different compared to the positive control setup comprised of macrophages exposed to LPS alone (for* IL1-* *β*, *P* = 0.012 in all probiotic treatment groups; for* TNF-α*, *P* = 0.001 in all probiotic treatment groups). No significant difference was found between the positive control and LPS-induced macrophages administered with the MRS culture broth (Figure 8).

The present study assayed the filter-sterilized supernatant from the probiotic cultures to determine whether* Lactobacillus *spp. from dairy products produce and secrete metabolites with cytotoxic and anti-inflammatory effects. The results showed that all three probiotic supernatants generally follow the same trend with regard to their cytotoxic, proapoptotic, and anti-inflammatory effects. The results of the study strongly suggest that the increased cytotoxicity for HT-29 and HCT116 cells (Figures 3(c)-3(d)) may be associated with a molecular pathway involving an upregulation of the early apoptosis gene markers* cfos* and* cjun*. Although studies have reported on the apoptotic effects of different* Lactobacillus* species on certain cancer cells [18, 19], not much emphasis was placed on the molecular aspect of apoptosis signaling mechanisms, specifically in relation to the aforementioned genes.

The pH values of the three (3) filter-sterilized supernatants were found to be 6.23, 6.35, and 6.37 for Yakult, Nido3+, and Bear Brand, respectively, which were not much more acidic than the uninoculated MRS broth with pH of 6.5 that was used as the negative control. Thus, it can be said that the consequent upregulation of both* cfos* and* cjun* after exposure to the probiotic supernatants was not due to stresses brought about by very low extracellular pH. The apoptotic effects of the probiotic supernatants may be mediated either by the formation of the AP-1 transcription factor that activates the MAPK pathway to induce cell death, or by increasing the susceptibility of the cancer cells to the TRAIL-induced apoptosis [6, 29].

From the experimental results, it was also observed that there was a significant decrease in the expression of both proinflammatory cytokine genes,* IL1-* *β* and* TNF-α*, following exposure of LPS-treated PMA-differentiated macrophages to the different probiotic supernatants.* Lactobacillus* species have been known to secrete metabolites that attenuate the production of the aforementioned cytokines in macrophages induced with the endotoxin in LPS [30, 31].

Anti-inflammatory effects of certain species of* Lactobacillus, *including* L. johnsonii *and* L. casei, *have been evaluated and subsequently confirmed through a reduction in the levels of T-cell-derived proinflammatory cytokine levels, including those of* IL-1* *β* and* TNF-α*among many others, while concomitantly increasing the levels of the anti-inflammatory cytokines* IL-4 *and* IL-10 *in response to inflammatory agents, such as lipopolysaccharide (LPS) and Type II collagen [32, 33]. Moreover, since the anti-inflammatory effect of the treatments was determined in terms of a significant decrease in the number of mRNA copies, the said effect on cytokine production may be narrowed down directly to the transcriptional level, specifically the nuclear factor kappa enhancer binding protein (NF-*κ*B) pathway. This may be a consequence of certain factors present in the supernatant that potentially caused the inhibition of the NF-*κ*B transcription factor itself or the inhibition of the ubiquitin protein, which is necessary for signaling the eventual activation of the NF-*κ*B transcription factor [34]. These ultimately lead to the prevention of the pathway from producing mRNA transcripts of the cytokine genes.

## 4. Conclusion

The results of the study showed that spent probiotic culture media from* Lactobacillus* spp. isolated from Bear Brand, Nido, and Yakult did not have a significant cytotoxic effect on normal HDFn and THP-1 leukemia cells but were significantly cytotoxic for the HT-29 and HCT116 colon cancer cell lines, as evidenced by their respective IC_50_ values. The cytotoxic activity may be due to the demonstrated upregulation of the early apoptotic gene markers,* cfos* and* cjun*, which was significantly higher than in the negative control (*P* < 0.05). Furthermore, the spent probiotic culture media also exhibited anti-inflammatory effects on LPS-induced macrophage cells, as evidenced by the significant downregulation of the proinflammatory cytokine genes,* IL1-* *β* and* TNF-α* (*P* < 0.05). This is likely achieved through the inhibition of the NF-*κ*B pathway. These results provide strong support for the role of probiotic* Lactobacillus* spp. in cancer treatment and prevention of inflammation as a precursor to carcinogenesis.
